# Amyloidosis of the Heart: A Comprehensive Review

**DOI:** 10.7759/cureus.35264

**Published:** 2023-02-21

**Authors:** Urooj Imdad

**Affiliations:** 1 Neurosurgery, Royal Preston Hospital, Preston, GBR

**Keywords:** cardiac amyloidosis, cardiac magnetic resonance imaging, amyloid cardiomyopathy, cardiac nuclear imaging, mattr amyloidosis, senile amyloidosis, al amyloidosis, transthyretin amyloid cardiomyopathy

## Abstract

Cardiac amyloidosis is a progressive, infiltrative cardiomyopathy, whose types are based on various infiltrating amyloids, namely, light chains in primary amyloidosis, mutated transthyretin proteins in hereditary amyloidosis, and wild-type transthyretin proteins in senile amyloidosis. While cardiac amyloidosis has a non-specific presentation, the type-specific presentations may provide some clues to the diagnosis. While tissue biopsy remains the gold standard, other newer non-invasive methods can aid in the diagnostic approach for suspected cardiac amyloidosis. Various medications used to treat heart failure may lead to adverse outcomes in patients with cardiac amyloidosis. More research is needed to understand the adequate management and treatment of cardiac amyloidosis.

## Introduction and background

Amyloidosis is the term given to the infiltration of fibrils and insoluble proteins, which normally circulate in the plasma, to the extracellular tissue, thereby disrupting the normal functions of many organs and leading to a wide variety of disease manifestations based on the organs involved. Systemic amyloidosis can involve any organ system, including the cardiovascular, respiratory, and nervous systems. Amyloid deposits also accumulate in individual organs such as the heart, kidney, liver, eye, lung, blood vessels, and skin. The involvement of the heart leads to fatal clinical manifestations and poor prognosis due to the development of angina, arrhythmias, and congestive heart failure.

## Review

Types of amyloidosis

Amyloidosis is classified into different types based on the type of amyloid involved. Some of these types involve multiple organs and organ systems, while others only cause protein deposition in particular organs. The common types that preferentially involve the heart are amyloid light-chain (AL) (primary) amyloidosis, familial transthyretin amyloid (mATTR) (hereditary) amyloidosis, and wild-type ATTR (senile systemic) amyloidosis.

Primary Amyloidosis

Primary (AL; light chain) amyloidosis is the most widely recognized and common type of systemic amyloidosis. It mostly affects the heart and the kidneys [1]⁠. In primary amyloidosis, the plasma cells in the bone marrow produce Ig light chains or fragments of light chains. The predominance of lambda light chains is three times that of kappa light chains [2]. The⁠ deposition of amyloid in the heart causes necrosis of the cardiac myocytes [3]⁠. The myocardium is extensively involved in AL amyloidosis as the infiltration can involve atria, ventricles, valves, and cardiac blood vessels [4]⁠. AL amyloidosis affects approximately 5-12 people per million per year, but the incidence might be even higher, as suggested by autopsy studies [5]⁠.⁠

Hereditary Amyloidosis

Hereditary (mATTR; familial) amyloidosis is caused by the deposition of misfolded mutated transthyretin protein, a transport protein normally produced by the liver and commonly found in the serum. The *TTR *gene for transthyretin is located on chromosome 18q12.1. It has five introns and four exons with more than 100 single nucleotide polymorphisms (SNPs) encoding the mutant *TTR *gene and over 80 pathogenic mutations, 45 of which involve the heart [6,7]⁠. The mutations follow an autosomal dominant pattern of inheritance, hence, equally affecting both genders and clustering into ethnic and/or geographic populations. The clinical presentation varies widely based on mutation type, gene penetrance, age of onset, and prognosis. For those with cardiac amyloidosis, patients usually show heart failure symptoms in their 50s [8]⁠. The most common hereditary cardiac amyloidosis is caused by *Val122Ile* mutation [9]⁠.

Age-Related Amyloidosis

Age-related (wtATTR; senile) amyloidosis is a result of tissue infiltration with the wild-type transthyretin protein. Unlike AL or mATTR amyloidosis, patients affected with wtATTR amyloidosis present much later in life, primarily over the age of 65 years, and it is seen predominantly in males at a ratio over 20:1 compared to females [10]⁠. In autopsy studies of patients aged 80 years or more, about 25% of the prevalence of wtATTR deposition in atria or left ventricle was found, suggesting histologically significant, but often not clinically significant, cardiac amyloidosis in people over the age of 80 years [11]⁠. Senile cardiac amyloidosis has a better prognosis than AL amyloidosis with 75 months of median survival [3]⁠. Considering the rise in the octagenarian population and comparably better prognosis, senile amyloidosis can become the most common type of cardiac amyloidosis over the next two decades.

Clinical manifestation

Cardiac amyloidosis does not have specific symptoms. The amyloid infiltration replaces the normal myocytes and fills the interstitium, causing changes at the intracellular and intercellular levels. The involved myocardium becomes stiff and non-compliant [12]⁠. Due to variable amyloid deposition, symptoms are based on the involvement of structural components and the extent of amyloid deposition in the heart. Cardiac amyloidosis may be asymptomatic or manifest as restrictive cardiomyopathy, congestive heart failure, valvular heart disease, coronary artery disease, arrhythmias, tamponade, ischemic stroke, syncope, and/or sudden death [1,13,14]⁠.

Type-specific presentation

AL Amyloidosis

Patients typically present at ≥40 years of age [15]⁠. If untreated, AL cardiac amyloidosis has a poor prognosis [16]⁠. AL amyloidosis can present as nephrotic syndrome, peripheral neuropathy, purpura, angina, claudication, and bleeding diathesis. It may also present with an enlarged liver or tongue. Cardiac involvement may be the predominant presentation in up to 30% of the patients [17]⁠.⁠ However, isolated cardiac involvement is rare [1]⁠. Capillary involvement presenting as periorbital purpura in a patient with heart failure strongly suggests AL amyloidosis.

AL amyloidosis of the cardiac conduction system often causes dysfunction of the His-Purkinje system [18]. High-degree atrioventricular block and symptomatic sinus node dysfunction are uncommon in AL amyloidosis [19]⁠. His-ventricular interval prolongation is common and is an independent predictor of sudden death in such patients [18]⁠. However, it may not be apparent when presenting with a narrow QRS complex. Amyloidosis can predispose to atrial thrombi by causing electrical and mechanical dysfunction of the atria during sinus rhythm [20]⁠.

mATTR Amyloidosis

The age of presentation varies according to mutation [15]⁠. *Val122Ile *mutation, seen in the African American and Afro-Caribbean populations, is characterized by severe progressive heart failure in patients over 50 years of age [9]⁠. *Val30Met *mutation is common in Portugal, northern Sweden, and Japan and often presents with lower extremity sensorimotor peripheral neuropathy in addition to cardiac involvement [21,22]⁠. Other less common mutations are associated with severe cardiomyopathy and minimal neuropathy [23,24]⁠.

Compared to AL amyloidosis, familial amyloidosis has a milder disease profile and a better prognosis [21,22,25]⁠. Progressive conduction system disease is common and often requires the implantation of a pacemaker.

wtATTR Amyloidosis

Patients present at the age of 60 years or above (commonly over 70 years) [15]⁠. Similar to familial amyloidosis, senile amyloidosis also carries a better disease profile and prognosis than AL amyloidosis [10]⁠. It also causes progressive conduction system disease, requiring pacemaker implantation.

Amyloid deposits in the atria are mostly derived from the atrial natriuretic peptide, while those in the ventricle are mostly derived from wtATTR. In most cases, these deposits hold no clinical significance⁠. However, atrial amyloid deposits increase the risk of atrial fibrillation [26]⁠.

Physical examination

Cardiac amyloidosis predominantly presents with restrictive cardiomyopathy leading to signs of diastolic heart failure. Atrial fibrillation and right-sided heart failure are common. Microvascular involvement can manifest as anginal chest pain, and autonomic dysfunction can cause syncope. In addition to cardiac findings, patients may also have hepatomegaly, ascites, periorbital purpura, and orthostatic hypotension.

Diagnostic approach

Tissue biopsy is the gold standard for the diagnosis of any amyloidosis. Because the signs and symptoms of amyloidosis are non-specific, histologic analysis of a biopsy specimen is required for a definitive diagnosis. However, there has been a shift toward non-invasive methods to diagnose cardiac amyloidosis. The non-invasive methods include electrocardiography (ECG), echocardiography with Doppler mode, chest radiography, cardiac MRI, and nuclear imaging. Many of these can be included in the diagnostic approach for suspected cardiac amyloidosis.

Electrocardiogram

In AL cardiac amyloidosis, the most common ECG abnormalities are low voltage (≤1 mV in all unipolar chest leads or ≤0.5 mV in all bipolar limb leads) and a pseudo-infarct pattern [27]⁠. These abnormalities are less common in other types of cardiac amyloidosis [15,16,22,27]⁠.

Cardiac Biomarkers

Cardiac biomarkers are sensitive markers of cardiac diseases. Troponin and N-terminal-pro-brain natriuretic peptide (NT-pro-BNP) are often abnormal in amyloidosis. However, the data on the use of biomarkers for diagnosis is insufficient, especially in ATTR amyloidosis. In contrast, the AL amyloidosis staging system has incorporated troponin and NT-pro-BNP levels [28,29]⁠. Troponin levels are associated with poor prognosis [30]⁠.

Echocardiography

Echocardiography shows a range of diastolic abnormalities along with ventricular wall thickening. Classic echocardiography findings include thick-walled ventricles with a granular or *sparkling* appearance, small left ventricular chamber volume, atrial enlargement, valvular thickening, thickened interatrial septum, and signs of increased filling pressures (dilated vena cava, pericardial effusion) [31-35]⁠. The echocardiographic criterion, decided by an international panel of experts in amyloid disease, identifies the involvement of the heart in patients with primary systemic amyloidosis if the interventricular septal thickness is more than 12 mm without aortic valvular disease or significant systemic hypertension [36]⁠.

Echocardiography is the most helpful imaging modality for identifying and monitoring cardiac amyloidosis. However, it is unable to differentiate between the types of cardiac amyloidosis [22]⁠. As echocardiograph findings of cardiac amyloidosis mimic hypertensive and hypertrophic cardiomyopathy, the diagnosis of cardiac amyloidosis is often delayed.

The combination of echocardiography findings of left ventricular thickening and ECG abnormality of low voltage has been reported to be highly specific (91-100%) and sensitive (72-79%) for cardiac amyloidosis [37,38]⁠.

Echocardiographic strain imaging is a non-invasive and reliable method for assessing and quantifying myocardial mechanical function and dysfunction. In longitudinal left ventricular strain, impairment is present even when the left ventricular ejection fraction is within the normal range [39]⁠. In cardiac amyloidosis, the change in strain follows a specific pattern. There is severe impairment of basal longitudinal strain, but the strain in apical segments is preserved. The apical sparing pattern is predictive of a better prognosis [40]⁠.

Cardiac Magnetic Resonance Imaging (CMR)

CMR offers high-resolution three-dimensional imaging of the myocardial border, walls, and cavities. It can precisely quantify ventricular volumes, wall thickness, and mass. Although echocardiography confers better temporal resolution, CMR is far superior for directly identifying amyloid infiltration using late gadolinium enhancement (LGE) imaging.

There is a distinctive pattern of late enhancement of the left ventricle on CMR with gadolinium contrast, even in patients with normal left ventricular wall thicknesses. This differentiates cardiac amyloidosis from other causes of cardiomyopathy and makes CMR LGE more sensitive for cardiac amyloidosis than echocardiography [41,42]⁠. Studies of the predictive value of CMR LGE for cardiac amyloidosis have found sensitivities of up to 88% and specificities of up to 92% [43-45]⁠.

Gadolinium contrast is relatively contraindicated in patients with moderate-to-severe kidney disease. Gadolinium contrast administration is associated with nephrogenic systemic fibrosis, a rare, severe, and potentially fatal syndrome [46]⁠. As many patients with suspected cardiac amyloidosis also have kidney disease, CMR LGE is used cautiously for diagnostic purposes in these patients.

Nuclear Scintigraphy

Nuclear scintigraphy is a promising diagnostic modality for the detection of cardiac amyloidosis. Among the nuclear imaging tracers that have been researched, technetium-phosphate derivatives such as 99mTechnetium-3,3-diphosphono-1,2-propanodicarboxylic acid (99mTc-DPD), 99mTechnetium-pyrophosphate (99mTc-PYP), and 99mTechnetium-hydroxymethylene diphosphonate (99mTc-HMDP) have been studied extensively for this purpose. A systematic review and meta-analysis assessing the diagnostic performance of CMR and nuclear scintigraphy showed that nuclear scintigraphy had high sensitivity (82%) and specificity (98.9%) against histology from any organ. Unlike CMR, it was able to differentiate ATTR amyloidosis from AL amyloidosis (sensitivity: 90.9-91.5%; specificity: 88.6-97.1%) [47].

Histopathology

While CMR findings can support the diagnosis of amyloid cardiomyopathy, and nuclear imaging is sensitive for diagnosing ATTR amyloidosis of the heart, a definitive diagnosis requires confirmation through tissue (myocardial or other) biopsy and histology demonstrating a positive Congo red stain with apple-green birefringence under polarized light. Cardiac amyloidosis is diagnosed if one or both of these conditions are met, namely, a positive endomyocardial biopsy or a positive non-cardiac biopsy in a patient with clinical, laboratory, or echocardiographic evidence consistent with amyloidosis [36]⁠. An endomyocardial biopsy is not recommended in a patient with a non-invasive appearance of cardiac amyloidosis but a positive biopsy for amyloid from a non-cardiac site.

Management

Symptomatic cardiac amyloidosis is managed by treating heart failure. Therapy for heart failure secondary to cardiac amyloidosis is different from that of systolic or diastolic heart failure. Loop diuretics are the first-line treatment for heart failure symptoms in cardiac amyloidosis and can be used in conjunction with aldosterone antagonists. Beta-blockers and calcium channel blockers seem to be poorly tolerated and show no proven benefit for cardiac amyloidosis patients. Angiotensin-converting enzyme inhibitors and angiotensin receptor blockers can lead to hypotension in cardiac amyloidosis and are typically avoided. Digoxin avidly binds to amyloid fibrils, which can precipitate digoxin toxicity in these patients.

Orthostatic hypotension can be alleviated with compression stockings and the alpha-1 blocker midodrine [3,17]⁠. In patients with conduction disease, pacemaker insertion may be required. Sudden cardiac death is a common outcome of patients with AL cardiac amyloidosis. Often, the cause of death is electromechanical dissociation instead of primary arrhythmias. The role of implantable cardioverter-defibrillator placement in cardiac amyloidosis is not clear. Cardiac transplantation in amyloidosis is a controversial topic because of the possible recurrence in the donor’s heart. Post-transplant outcomes in amyloidosis patients seem to be worse than post-transplant outcomes in patients with other causes [48]⁠.

Newer systemic therapies aim to correct the misfolding of amyloid proteins. Some patients with systemic AL amyloidosis may be treated with chemotherapy and/or autologous hematopoietic cell transplant. Tafamidis, a transthyretin stabilizer, has shown reduced mortality in patients with ATTR cardiomyopathy [49]. A hepatic transplant may also prove to be curative in select patients with mATTR amyloidosis. Patients with advanced amyloid cardiomyopathy may be treated with cardiac and hepatic transplantation [50].

## Conclusions

Cardiac amyloidosis is a rare disease with a poor prognosis. With its non-specific presentation, the diagnosis is often overlooked. Echocardiography is the primary modality for diagnosis. CMR and nuclear imaging have the potential for earlier diagnosis and risk evaluation of cardiac amyloidosis. CMR offers high-resolution myocardial tissue imaging, and nuclear imaging can distinguish between the types of cardiac amyloidosis. Treatment of cardiac amyloidosis in a patient with heart failure symptoms requires a careful selection of medicines. Other treatment options for cardiac amyloidosis need further research.
